# Early Diagnosis of Streptococcal Toxic Shock Syndrome With Abdominal Computed Tomography: A Case Report

**DOI:** 10.7759/cureus.90348

**Published:** 2025-08-17

**Authors:** Ryo Kurokawa, Mariko Kurokawa, Soma Onoda, Yusuke Onozato, Akihito Nakajima, Tomoki Wada, Osamu Abe

**Affiliations:** 1 Department of Radiology, The University of Tokyo, Tokyo, JPN; 2 Department of Emergency and Critical Care Medicine, The University of Tokyo, Tokyo, JPN

**Keywords:** computed tomography, group a streptococcus, gynecologic procedure, peritonitis, streptococcal toxic shock syndrome

## Abstract

Streptococcal toxic shock syndrome (STSS) is a rapidly progressive and highly lethal infection caused by group A streptococcus, in which early diagnosis remains challenging due to nonspecific initial symptoms. We report a case of a 37-year-old woman who developed severe abdominal pain 24 hours after outpatient cervical polypectomy, with progression to septic shock at 48 hours post-procedure, presenting with hypotension, diffuse erythema, leukopenia, acute kidney injury, and elevated inflammatory markers. Contrast-enhanced abdominal computed tomography (CT) demonstrated pelvic-predominant peritoneal thickening, uniform small-bowel wall thickening with serosal thickening, and mesenteric vascular engorgement, findings suggestive of toxin-mediated peritonitis rather than primary enteritis. Based on clinical presentation and CT findings, invasive group A streptococcal infection with STSS was strongly suspected, prompting immediate treatment with meropenem plus clindamycin and hemodynamic support before obtaining the blood culture result. The next day, blood cultures confirmed *Streptococcus pyogenes*, establishing the diagnosis of STSS. The patient achieved hemodynamic stability within 24 hours of appropriate antibiotic therapy, ultimately recovering without major disability, and was discharged on Day 44. This case demonstrates that contrast-enhanced CT can provide crucial diagnostic information for early STSS recognition, particularly the characteristic pattern of pelvic-centered peritonitis with small bowel involvement, and when STSS is strongly suspected based on clinical history and CT findings following minor gynecologic procedures, clinicians should consider initiating β-lactam plus clindamycin therapy without waiting for culture confirmation to optimize patient outcomes.

## Introduction

Streptococcal toxic shock syndrome (STSS) is a rapidly progressive and highly lethal severe infection caused by toxin-producing strains of group A β-hemolytic streptococci (*Streptococcus pyogenes*) and other related organisms. In the United States, invasive group A streptococcus (iGAS) infection occurs at a rate of 3.8 cases per 100,000 population annually, with approximately 4% of these cases (approximately 0.15 per 100,000 population annually) developing STSS, which carries a mortality rate of 38% [1]. In Japan, iGAS infections have increased dramatically following the severe acute respiratory syndrome coronavirus 2 pandemic, representing an important public health concern [2]. In STSS, pyrogenic exotoxins acting as superantigens induce rapid-onset shock and multiorgan failure through the release of inflammatory cytokines [3]. Nonspecific symptoms such as fever, chills, myalgia, vomiting, and diarrhea are followed by the rapid development of hepatic failure, renal failure, acute respiratory distress syndrome, disseminated intravascular coagulation, soft tissue inflammation, generalized erythematous rash, and central nervous system symptoms within 24-48 hours, leading to critical illness. Under the Infectious Diseases Control Law in Japan, physicians must report STSS cases that meet criteria for shock symptoms and >2 of the following conditions: hepatic failure, renal failure, acute respiratory distress syndrome, disseminated intravascular coagulation, soft tissue inflammation, generalized erythematous rash, central nervous system symptoms, or detection of β-hemolytic streptococci from typically sterile sites [2]. Early diagnosis of STSS is challenging due to its nonspecific initial presentation and rapid progression. However, while delayed diagnosis is associated with mortality rates reaching 38%, accumulating evidence demonstrates that concurrent use of clindamycin, a toxin-suppressing antimicrobial agent, when STSS is suspected, can significantly reduce mortality [4,5]. In this article, we report a case of STSS triggered by cervical polypectomy, in which contrast-enhanced abdominal computed tomography (CT) was useful for early diagnosis and prompt therapeutic intervention, resulting in a favorable clinical outcome.

## Case presentation

The patient was a 37-year-old woman who underwent cervical polypectomy at a local clinic on Day 1. An antimicrobial vaginal suppository was inserted, with no other procedures or medications administered. On Day 2, she developed persistent abdominal pain (numerical rating scale: 8/10) extending from the left lower abdomen to the umbilical region, prompting emergency medical consultation. No evident abnormal findings responsible for acute abdomen, including STSS, were identified on contrast-enhanced CT, and blood tests were considered non-pathological, requiring no emergent intervention. She was discharged home after temporary improvement following intravenous acetaminophen administration.

**Figure 1:**
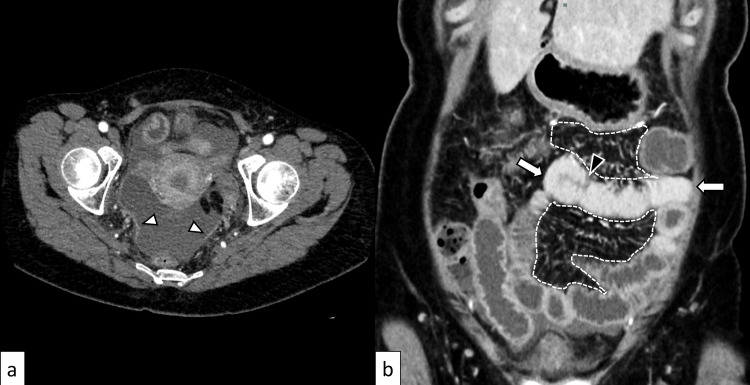
Contrast-enhanced abdominal computed tomography Peritoneal thickening with contrast enhancement is observed predominantly in the pelvis (a, white arrowheads). The small bowel demonstrates apparently uniform wall thickening resulting from obliteration of spaces between Kerckring's folds due to mucosal and villous swelling (b, arrows). Serosal swelling is also present (b, black arrowhead), along with dilation of adjacent mesenteric vessels (b, dotted lines).

On Day 3, she returned due to worsening pain. Although conscious and alert, she presented with shock, including a systolic blood pressure of 57 mmHg, a heart rate of 115 beats per minute, and a body temperature of 36.8°C. Diffuse erythema extending from the neck to the thighs and generalized abdominal tenderness were observed, although muscle guarding and rebound tenderness were minimal. Transthoracic echocardiography revealed a left ventricular ejection fraction of approximately 40% and inferior vena cava collapse, suggesting hypovolemia. Laboratory findings were as follows: white blood cell count: 2.4 × 10³/µL (reference range: 3.3-8.6 × 10³/µL), platelet count: 16.2 × 10⁴/µL (reference range: 15.8-34.8 × 10⁴/µL), C-reactive protein: 25.7 mg/dL (reference range: <0.3 mg/dL), creatinine: 2.09 mg/dL (reference range: 0.46-0.79 mg/dL), and total bilirubin: 0.7 mg/dL (reference range: 0.4-1.5 mg/dL). Arterial blood gas analysis revealed metabolic acidosis with pH 7.368 and HCO₃⁻ 16.6 mmol/L (reference range: 22-26 mmol/L). Contrast-enhanced abdominal CT was performed again, and a diagnostic radiologist confirmed extensive peritonitis centered in the pelvis (Figure 1). Serosal inflammation in the small bowel with vascular engorgement in the mesentery was considered peritonitis involving the small bowel rather than infectious enteritis. Combined with the clinical course, iGAS infection with STSS was strongly suspected.

In the emergency department, piperacillin/tazobactam 4.5 g was administered intravenously as empirical therapy, followed by prompt initiation of continuous norepinephrine and vasopressin infusions for blood pressure support. Endotracheal intubation and mechanical ventilation were required for respiratory support. Hydrocortisone 200 mg was administered intravenously, considering relative adrenal insufficiency. As STSS was suspected based on the clinical course and contrast-enhanced CT findings, antibiotic therapy was changed to meropenem 1 g every 12 hours plus clindamycin 600 mg every eight hours after intensive care unit admission. Hemodynamic stability was achieved from the following day.

On Day 4, blood cultures (2/2 sets), vaginal discharge, and urine specimens collected on Day 3 yielded hemolytic streptococci (*Streptococcus pyogenes*). Escherichia coli was also isolated from the vaginal discharge specimen. These findings, combined with severe hemodynamic instability, renal dysfunction, and diffuse erythema, confirmed the diagnosis of STSS. On day 6, it was revealed that *Streptococcus pyogenes* had also been detected in vaginal secretions collected before polypectomy at a local clinic on day 1. On day 6, it was revealed that *Streptococcus pyogenes* had also been detected in vaginal secretions collected before polypectomy at the referring clinic on Day 1, though this positive result became available only after the procedure, and the polypectomy had proceeded because there were no clinical signs of active genital-tract infection. Given that *Streptococcus pyogenes* had been detected in vaginal secretions prior to polypectomy and that the peritonitis observed on contrast-enhanced CT showed a predominantly pelvic distribution, the route of infection was considered to be ascending infection associated with the procedure. Notification to the infectious disease surveillance system was completed in accordance with legal requirements.

The patient was extubated on Day 12 and transferred to the general ward on Day 15. Following the transfer, transient drug eruption and fever occurred, which resolved with discontinuation of the suspected causative drugs and oral prednisolone therapy. On Day 44, renal function had nearly recovered, and the skin rash had resolved, allowing for hospital discharge.

## Discussion

We report a case of STSS in a 37-year-old woman only 48 hours after an outpatient cervical polypectomy. Contrast-enhanced abdominal CT disclosed diffuse pelvic peritonitis with small intestine involvement, prompting empiric broad-spectrum antibiotics plus toxin-suppressive clindamycin and early hemodynamic support, and the patient survived without major disability.

Worldwide incidence of invasive group A streptococcal infection is rising; the United States reports ≈3.8 cases/100,000 persons annually, of which 4 % progress to STSS, while Japan documented an unprecedented post‑pandemic surge in 2024-25 [1,2]. Case‑fatality for STSS in adults still exceeds 30 % despite modern critical care. Current STSS-specific guidance recommends inpatient treatment with beta-lactam therapy plus clindamycin to blunt exotoxin production, in addition to aggressive organ-supportive therapy [6]. A 2022 systematic review of 1,059 patients confirmed that adjunctive clindamycin independently halves mortality (risk ratio 0.48, 95% confidence interval 0.30-0.77) [5]. Evidence for the use of intravenous immunoglobulin remains conflicting. A 2023 observational study in iGAS infection failed to show a survival benefit, underscoring the need for individualized use in the sickest patients only [7].

Although imaging is not explicitly addressed in current STSS management, our case and recent radiological literature suggest that it is pivotal. Multiple reports exist of iGAS infections resembling our case, in which female patients complained of malaise the day after undergoing outpatient minor gynecologic procedures and rapidly developed STSS [8-10]. A common lesson learned from these previous reports is that clinical and CT findings were initially interpreted as infectious enteritis or nonspecific peritonitis, and since iGAS infection was not strongly suspected, administration of clindamycin, which significantly reduces STSS mortality, was not initiated until after culture results were obtained. Available CT images from previous reports demonstrated findings similar to our case: peritoneal thickening with associated small bowel wall thickening lacking a layered structure, relatively uniform enhancement and dilated mesenteric vessels [8,9]. In contrast to infectious enteritis, small bowel involvement in peritonitis results from external inflammatory spread, causing serosal inflammation and increased arterial blood flow. This leads to increased arterial perfusion throughout the bowel tissue, resulting in vascular engorgement and tissue swelling. Mucosal and villous swelling obliterates the spaces between Kerckring's folds, producing an appearance of uniform wall thickening (serosal swelling also occurs). CT imaging reflects these pathophysiological changes, with serosal enhancement and swelling representing characteristic features of small bowel involvement with peritonitis.

## Conclusions

Timely contrast-enhanced CT can be a decisive adjunct for the early diagnosis and source identification of post-procedural STSS, thereby expediting guideline-concordant antimicrobial therapy and hemodynamic resuscitation. In patients who present with disproportionate pain or systemic toxicity after minor gynecologic interventions, clinicians should maintain a high index of suspicion, and when STSS is strongly suspected based on clinical history, symptoms, and CT imaging findings suggestive of peritonitis centered in the pelvis with small bowel involvement, consideration should be given to initiating β‑lactam plus clindamycin therapy without waiting for culture confirmation. Future prospective studies should formally evaluate imaging‑triggered pathways in STSS to refine recommendations and improve outcomes in this high‑mortality infectious syndrome.
